# Development of a burst wave lithotripsy system for noninvasive fragmentation of ureteroliths in pet cats

**DOI:** 10.1186/s12917-023-03705-1

**Published:** 2023-09-02

**Authors:** Adam D. Maxwell, Ga Won Kim, Eva Furrow, Jody P. Lulich, Marissa Torre, Brian MacConaghy, Elizabeth Lynch, Daniel F. Leotta, Yak-Nam Wang, Michael S. Borofsky, Michael R. Bailey

**Affiliations:** 1 Department of Urology, University of Washington School of Medicine, Seattle, WA USA; 2 Center for Industrial and Medical Ultrasound, Applied Physics Laboratory, University of Washington, Seattle, WA USA; 3 Department of Veterinary Clinical Sciences, University of Minnesota, St. Paul, MN USA; 4 Department of Urology, University of Minnesota, Minneapolis, MN USA

## Abstract

**Background:**

Upper urinary tract stones are increasingly prevalent in pet cats and are difficult to manage. Surgical procedures to address obstructing ureteroliths have short- and long-term complications, and medical therapies (*e.g.,* fluid diuresis and smooth muscle relaxants) are infrequently effective. Burst wave lithotripsy is a non-invasive, ultrasound-guided, handheld focused ultrasound technology to disintegrate urinary stones, which is now undergoing human clinical trials in awake unanesthetized subjects.

**Results:**

In this study, we designed and performed in vitro testing of a modified burst wave lithotripsy system to noninvasively fragment stones in cats. The design accounted for differences in anatomic scale, acoustic window, skin-to-stone depth, and stone size. Prototypes were fabricated and tested in a benchtop model using 35 natural calcium oxalate monohydrate stones from cats. In an initial experiment, burst wave lithotripsy was performed using peak ultrasound pressures of 7.3 (*n* = 10), 8.0 (*n* = 5), or 8.9 MPa (*n* = 10) for up to 30 min. Fourteen of 25 stones fragmented to < 1 mm within the 30 min. In a second experiment, burst wave lithotripsy was performed using a second transducer and peak ultrasound pressure of 8.0 MPa (*n* = 10) for up to 50 min. In the second experiment, 9 of 10 stones fragmented to < 1 mm within the 50 min. Across both experiments, an average of 73–97% of stone mass could be reduced to fragments < 1 mm. A third experiment found negligible injury with in vivo exposure of kidneys and ureters in a porcine animal model.

**Conclusions:**

These data support further evaluation of burst wave lithotripsy as a noninvasive intervention for obstructing ureteroliths in cats.

## Background

Upper urinary tract stones in cats are a significant cause of morbidity and mortality [1, 2] The vast majority of these stones (87–98%) are calcium-based [3–5]. There is no protocol for medical stone dissolution of calcium-based stones, and medical management with intravenous fluid therapy and drugs to facilitate obstructing stone passage are effective in only a small proportion of patients (13%) [1, 6]. In the remaining cases, placement of a subcutaneous ureteral bypass (SUB) device or ureteral stent is recommended to relieve obstruction [7]. However, these procedures carry a 6–18% perioperative mortality rate and a 29–56% risk of long-term complications such as device occlusion, urinary tract infection, and, in the case of stents, lower urinary tract signs without infection [4, 8–11]. The high costs and specialized techniques required for placement of SUB devices or stents also limit availability of these options. For these reasons, new minimally invasive techniques and procedures are sought to improve the efficacy, safety, and availability of treatments for feline ureteroliths.

Minimally invasive techniques such as shock wave lithotripsy (SWL) [12] and endoscopic laser lithotripsy [13] are the primary interventions for stones in humans. These methods are not available for treating ureteroliths in cats, as feline calcium oxalate uroliths are relatively resistant to fragmentation by SWL and the feline ureter is too small for laser lithotripsy instrumentation [7, 14]. Burst wave lithotripsy (BWL) is a new noninvasive approach to fragment urinary tract stones based on focused ultrasound technology. This method uses a focused ultrasound transducer to apply short harmonic bursts of ultrasound to a stone to produce cyclic stressing that leads to fractures and fragmentation [15]. A BWL system consists of a small electronic pulser and therapy transducer that can be coupled to the skin of a patient by a thin layer of ultrasound gel. An ultrasound imaging probe incorporated with the therapy head allows detection and localization of a stone, using custom algorithms to enhance detection of calcifications [16, 17]. This technology has progressed from conception to human clinical trials over the last 8 years [18, 19]. BWL has been demonstrated to fragment a number of different stone compositions, including calcium oxalate stones, in preclinical and human studies [19, 20], without producing any significant injury or complications [20, 21]. In a preclinical study using calcium oxalate monohydrate stones (known to be resistant to SWL) implanted in the kidneys of pigs, stones were noninvasively fragmented by BWL, with 88% of the resulting fragments smaller than 2 mm [20]. No injury was found to the kidney parenchyma and only mild hemorrhagic injury to the collecting space lining. The fine control of acoustic parameters such as amplitude and frequency has been demonstrated to have a number of benefits, one of which is the control of the size of fragments generated from the stone during the procedure [19]. For instance, 170 kHz ultrasound bursts produce fragments in artificial stones up to 4 mm, while 800 kHz ultrasound produce fragments approximately 0.6 mm in size. While the normal luminal diameter of the feline ureter is estimated at 0.3–0.4 mm, the average diameter of an obstructed ureter is 3.2–3.5 mm, and it can dilate up to 11 mm [2, 8, 22]. Furthermore, the typical diameter of ureteral stents in cats is 1 mm, and only 2% of ureteroliths are ≤ 1 mm in cats [4, 11, 22]. BWL can therefore be designed to noninvasively fragment feline ureteroliths to pieces small enough to pass through the cat ureter, while minimizing the risks of injury and complications associated with surgery.

In this paper, we describe the design and testing of a BWL system adapted to noninvasively treat ureteroliths in cats. These studies include analysis of the acoustic window and skin-to-stone depth in cats, design of electronics and a small ultrasound-guided transducer, demonstration of stone fracture on natural feline ureteroliths, and preliminary evaluation of potential soft tissue injury.

## Methods

**Fig. 1:**
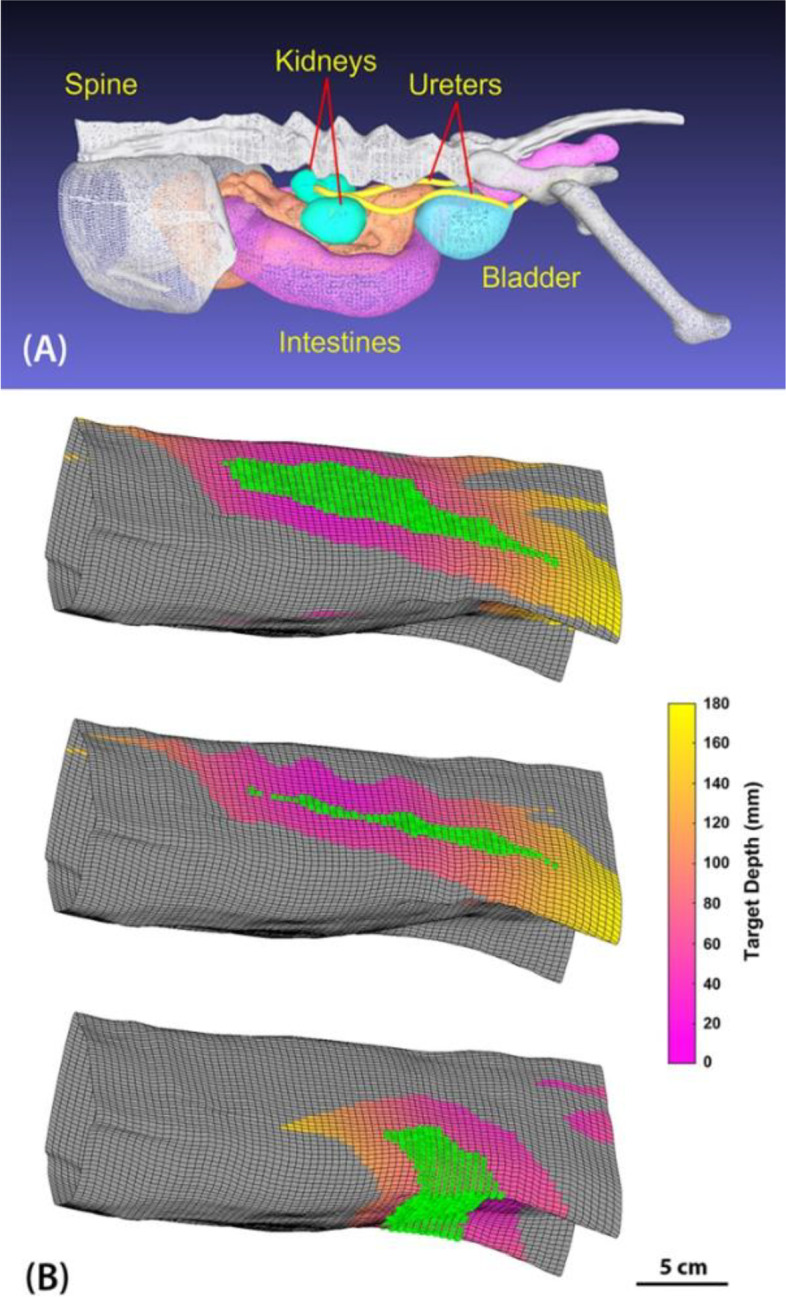
**A** Three-dimensional anatomic surface models derived from CT data, showing the urinary tract, bone structure, and intestinal tract in a cat. **B** Surface rendering of the skin, with green points indicating center positions of a 5-cm diameter transducer with a clear acoustic window to the left kidney, left proximal ureter, and urethra (top to bottom). The pink-yellow scale indicates the skin-to-stone depth at different points along the surface with a clear acoustic path to the stone

Burst wave lithotripsy uses a focused ultrasound transducer to apply high-amplitude pressure pulses to a stone to fragment it. However, human systems that have been designed for this application are not ideal for treating cats, as the therapy devices are too large, must focus deep (5-10 cm) from the skin surface, incorporate imagers for abdominal imaging in humans, and are designed to fragment stones into ≤ 2 mm fragments (small enough for humans to pass but not necessarily for cats). To address these differences, we have developed an initial prototype system designed for use in cats, operating at 650 kHz with a smaller transducer to treat at shallower depths than humans and adapted for a veterinary ultrasound imager. Computed tomography and ultrasound images of cats with ureteroliths were analyzed to determine the optimal acoustic window and the appropriate dimension for the transducer. The transducer is designed to target ureteroliths while avoiding bone and other vital structures (Fig. 1).

### Anatomic analysis

Image analysis from B-Mode ultrasound images and computed tomography (CT) 3-dimensional data were performed to assess an approximate focal depth and acoustic window for design of the BWL transducer. The acoustic window is the anatomic aperture where ultrasound can pass through soft tissue to the focal point without encountering reflective obstructions such as bone or gas in the intestines or lung. This window limits the dimensions of the ultrasound device placed on the skin.

Images were captured from previous patients with ureteral stones. Skin-to-stone distance was measured in ten cats manually from ultrasound images. CT data for one cat was manually outlined using custom software [23] to segment the skin surface, bone, kidneys, ureters, bladder and urethra, and small/large intestine as separate tissues. The outlines were converted to 3D surface models using custom software developed in MATLAB (MathWorks, Natick, MA). An acoustic window analysis was performed to determine the largest-diameter ultrasound transducer that could be applied without bone or intestines (which block ultrasound) intervening in any part of the beam path to a target location within the urinary tract. Target locations in the kidney, proximal and distal ureter, and urethra were assessed. The distances at these different locations from the transducer were also calculated as a function of the transducer position to determine optimal windows for therapy in each scenario.

### Device design and fabrication

Based on image analysis, a BWL transducer was designed using a circular piezoelectric transducer element and lens model [24]. The transducer beam profile was calculated using the Rayleigh integral [25, 26] and custom piezoelectric simulation software based on a version of the KLM model [27]. The frequency of the ultrasound was selected based on relative frequency to reduce stones to a size < 1 mm [19, 20].

The transducer was designed in a solid modeling program (Solidworks, Dassault Systemes, Vélizy-Villacoublay, France). The housing incorporating the focusing lens was produced on a resin-based 3D printer (3SP, Envisiontech Dearborn, MI) and the piezoelectric elements were bonded to the lens through a quarter-wavelength epoxy-aluminum oxide composite acoustic matching layer. A water bolus with latex rubber membrane was incorporated to the front of the transducer to couple the transducer to skin. Two fluid ports were installed to allow degassed water to be added to the space between the membrane and transducer for coupling. An RG-174 coaxial cable was soldered to the transducer element to apply voltage to produce ultrasonic vibration of the piezoelectric element.

A small electronic pulser was constructed to apply the voltage signal to the transducer and power it. The pulser was constructed based on a previous design to apply short, high amplitude sinusoidal voltage pulses [28]. The system used an FPGA based architecture to be able to rapidly reconfigure the output parameters as needed during testing. A passive electrical network was constructed to increase the voltage applied to the transducer and the power output.

The system was calibrated using a fiber optic probe hydrophone (FOPH2000, Leutenbach, Germany) in an open bath of degassed, deionized water [29]. The transducer was positioned facing the hydrophone probe tip and the peak pulse pressure values, focus location, and focus beamwidths were measured by scanning the probe tip while affixed to a 3-axis motorized positioner. The collected signals were output to an oscilloscope (DSOX3034, Keysight, Santa Rosa, CA) and deconvolved using custom software programmed in MATLAB.

### Testing on stones

To assess the capability of the transducer to fragment uroliths, the assembled BWL system was tested on natural stones. Calcium oxalate stones (100% monohydrate) were collected from open surgeries at University of Minnesota Veterinary Medical Center. The stone diameters were between 2–5 mm. Stone composition was analyzed by polarization microscopy and infrared spectroscopy and shipped in a dry state. The stones were rehydrated in water at least 48 h before experiments.

A small plastic holder was used to position each stone at the focus of the transducer in a degassed water bath. Each stone was held freely in a small depression in a molded synthetic gelatin mass at the center of the holder to mimic the surrounding tissue. The holder magnetically attached to the front of the transducer to automatically position the stone at the center of the focus.

Experiments were performed in two stages. A first experiment was conducted to compare three different pressure amplitudes for exposure and to optimize this parameter. Burst wave lithotripsy pulses were applied at focal pressure amplitudes of 7.3, 8.0, or 8.9 MPa with 20-cycle pulse duration at a rate of 10 pulses per second. Each stone was treated in 10-min intervals for up to 30 min total exposure. At each interval, the stone fragments were passed through serial sieves with 1.0-mm and 0.7-mm openings. The fragments larger than each size were weighted with an analytical balance and replaced in the holder for further treatment to determine the fraction of stone fragmented at each time point.

A second experiment was conducted with the pressure level showing the greatest efficacy (8.0 MPa) to confirm the consistency of the results with a second transducer. The BWL parameters were kept the same, but stones were treated for up to 50 min in 10-min intervals, which we considered clinically practical.

### In vivo evaluation in a porcine kidney and ureter

All procedures were approved by the University of Washington’s Institutional Animal Care and Use Committee. In the third experiment, 3-mm calcium oxalate monohydrate stones were soaked overnight before being implanted into the kidneys and ureters of four live pigs as described previously [30]. The pig was chosen due to our experience working with the pig model for stone comminution and expertise in evaluating potential injury from BWL in this model. Pigs were obtained from Progressive Swine Farms (Woodinville, WA), an approved swine provider to the University of Washington. Ties were placed on either side of the stone in the ureter to prevent movement of the stone during treatment. The procedure was performed in an open abdomen, with the transducer directly coupled to the kidney/ureter by degassed saline and placed over the stone. As the ultrasound imaging was unavailable at the site of animal studies, an audible clicking associated with the BWL bursts impacting the stone confirmed alignment and was used to realign the focus if the stone or transducer moved. Each stone was treated for 10 min at 8 MPa. After treatment, the location of the transducer was marked to ensure tissue was harvested from the correct location. After treatment, the pig was euthanized with an intravenous injection of sodium pentobarbitol (87 mg/kg, Euthasol ®, Virbac Inc., Westlake, TX) while still under anesthesia (1–3% isoflurane), according to AVMA guidelines. After euthanasia, the kidneys and ureter were harvested for gross evaluation before being processed for histological evaluation. Tissue sections taken from the treatment locations were stained with hematoxylin and eosin (H&E).

## Results

### Acoustic window analysis

Ten B-Mode ultrasound scans were analyzed to determine a range of skin-to-stone depths for obstructing ureteral stones. All stones were easily visualized by ultrasonography. The scans indicated depths ranging from 0.7 – 2.1 cm from the skin surface, with a mean of 1.3 cm.

CT analysis showed minimum skin-to-stone distances from 2.4 to 3.8 cm for seven locations (Table 1), somewhat greater than the range captured by ultrasonography. However, the distances calculated from CT do not take into account the compression of tissue typically applied by the probe to maintain contact during ultrasound imaging. The analysis indicated that all stone locations could be targeted with an open acoustic window for a transducer maximum size up to 6 cm. The available transducer positions and skin-to-stone depths for 3 stone locations for a 5-cm diameter transducer are shown in Fig. 1. The minimum acoustic window size is defined as the spatial distribution of allowed transducer center-points (green points in Fig. 1B).

**Table 1:** Skin-to-target depths and acoustic window minimum dimensions for a simulated transducer calculated from computed tomography images of a cat as a function of location

Position	Minimum Skin-to-Target Depth (cm)	Acoustic Window Size (cm^2^)
Left Kidney	2.5	59.0
Right Kidney	2.8	21.3
Left Proximal Ureter	3.2	16.6
Right Proximal Ureter	3.8	10.6
Left Ureterovesical Junction	2.6	133.1
Right Ureterovesical Junction	2.4	109.7
Urethra	2.9	82.2

### Transducer design

**Fig. 2:**
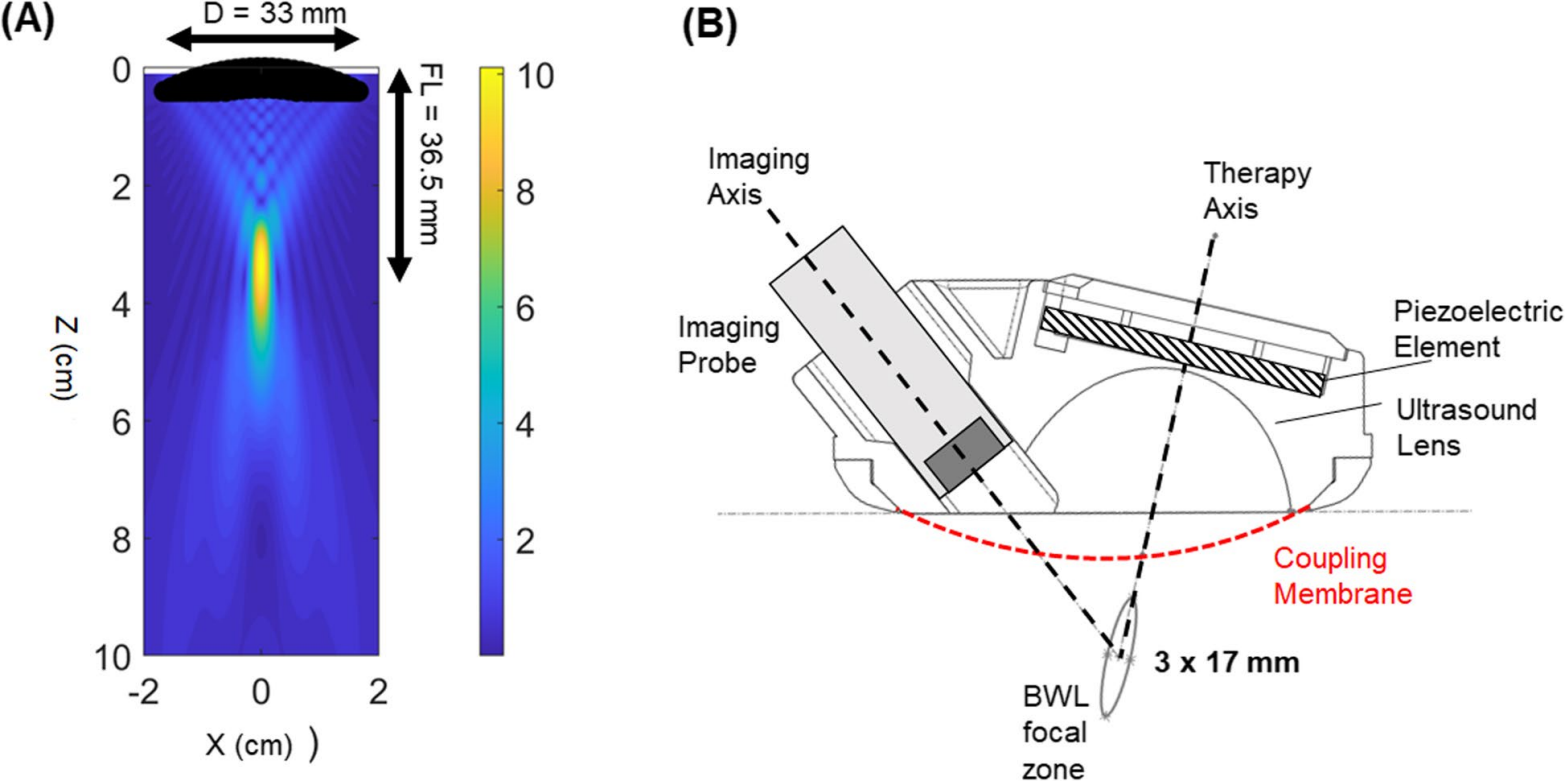
**A** Simulated ultrasound pressure field for the designed burst wave lithotripsy transducer. The pressure value corresponds to the relative pressure between the focus and surface of the transducer. **B** Cross sectional diagram of the transducer design, containing the piezoelectric element, acoustic lens, and off-axis ultrasound imaging transducer for therapy monitoring

**Fig. 3:**
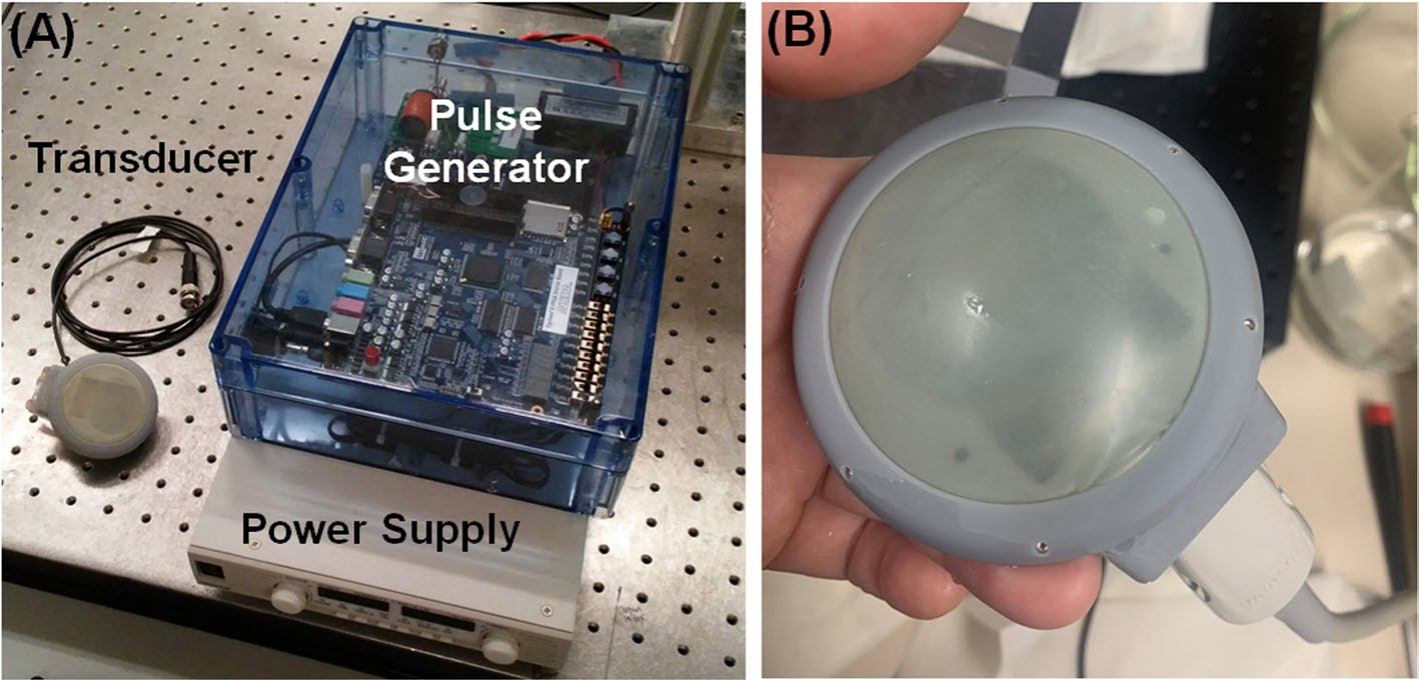
Design of a burst wave lithotripsy system for fragmentation of feline ureteroliths. **A** The fabricated transducer and pulse generator with power supply. **B** Close-up of the transducer applicating surface, using a rubber membrane covering filled with water between the membrane and transducer for flexible contact with the skin

The transducer was designed with a therapy aperture of 3.3 cm diameter, with a focal length of 3.7 cm. The transducer was constructed from a flat piezoceramic element bonded to a focusing lens through an acoustic matching layer. The distance of 3.7 cm focal length is from the apex of the concave lens to the center of the focus, which placed the front edge of the transducer approximately 1.6 cm from the focus when against the skin surface. To accommodate shallower skin-to-stone distances, a flexible membrane was fitted over the transducer surface. The gap between the membrane and transducer is filled with water to cause the membrane to expand and adjust the position of the focus relative to skin surface. Coupling gel is applied first to ensure good ultrasound transmission at the membrane-skin interface. A microconvex imaging transducer with bandwidth of 4–10 MHz (PVT712, Toshiba Medical, Otawara, Tochigi, Japan) was incorporated, obliquely aligned the therapy transducer (Fig. 2). With the combined therapy and imaging transducers in position, the overall aperture of the device was 5.0 cm (Fig. 3).

The transducer beam geometry was characterized under linear (low amplitude) acoustic output. The focal region was ellipsoidal with a lateral -6 dB beamwidth of 3 mm and an axial beamwidth of 17 mm. Hydrophone measurements recorded a peak negative pressure output of up to -8.9 MPa at the maximum driving voltage. Comparatively, human treatments have been performed at up to 7 MPa in situ pressure amplitude, suggesting sufficient pressure to fragment stones [18, 31, 32].

### Stone exposure

**Fig. 4:**
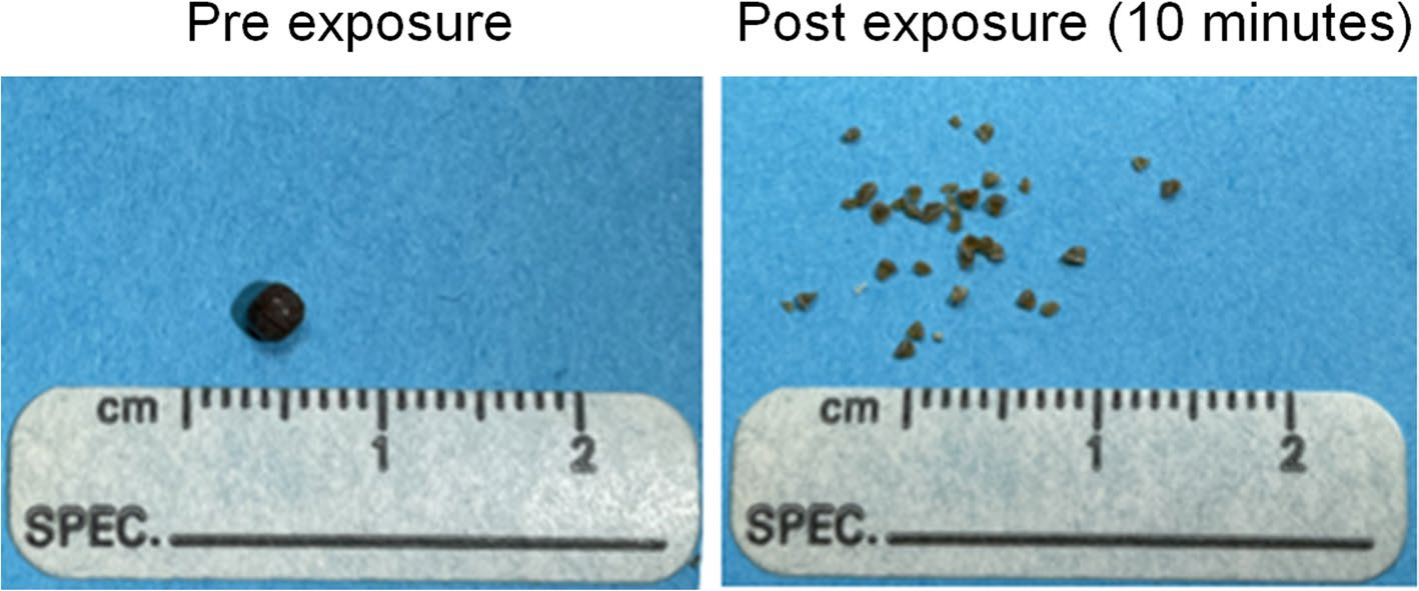
Example of a feline calcium oxalate urolith before (left) and after (right) 10 min of burst wave lithotripsy at 8.0 MPa pressure amplitude

**Fig. 5:**
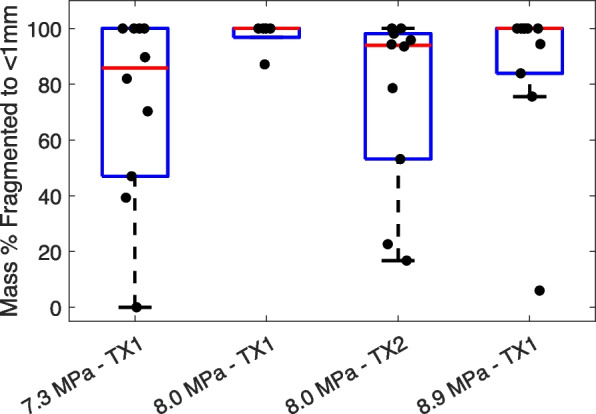
Boxplots showing the percentage of the mass of a stone fragmented to < 1 mm as a function of focal pressure and transducer for burst wave lithotripsy exposures up to 30 min. The black circles are individual data points for each stone. TX1 indicates the first transducer/experiment and TX2 indicates the second transducer/experiment

All stones were composed of 100% calcium oxalate monohydrate and had nearly spherical morphology. The stones were between 2–5 mm maximum dimension and had a mass between 4 – 61 mg (average mass 15–16 mg for each group). In the first experiment, the stones were exposed to either 7.3 MPa (*n* = 10), 8.0 MPa (*n* = 5), or 8.9 MPa (*n* = 10) peak ultrasound pressure in 10-min increments up to 30 min total exposure per stone. Figure 4 shows the average fragmented mass as a function of time for each of the 3 pressure levels. Depending on the pressure level administered, the average stone mass reduced to pieces smaller than 1 mm varied from 73 ± 32% to 97 ± 5%, and between 43 ± 28%—77 ± 19% reduced to fragments smaller than 0.7 mm after 30 min (Fig. 5). Of the 25 stones treated, 14 (56%) were completely reduced to fragments < 1 mm within 30 min of exposure. 2–3 stones in each group achieved complete fragmentation to < 1 mm pieces within 10 min. Only 2 of the treated stones showed no visible fragmentation over the 30-min period.

**Fig. 6:**
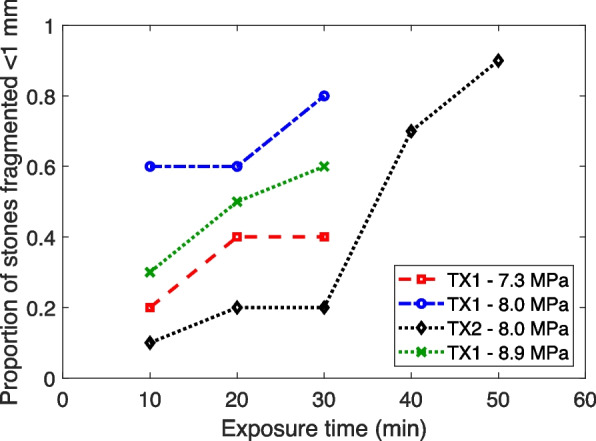
Proportion of stones completely fragmented to < 1 mm pieces as a function of total exposure time in the four treatment groups across two experiments. TX1 indicates the first transducer/experiment and TX2 indicates the second transducer/experiment

In a second experiment, a separate transducer and system were used to perform BWL on 10 stones in vitro at 8 MPa pressure amplitude for up to 50 min. In this experiment, 75 ± 31% of the stone mass was reduced to < 1 mm within 30 min (Fig. 5), but only 2 stones were completely fragmented at this point (1 of which completely fragmented within 10 min). However, by 50 min’ exposure time, 9 of 10 stones were completely fragmented in this experiment, and 97 ± 0.6% of the mass was < 1 mm (Fig. 6).

### In vivo evaluation in a porcine kidney and ureter

**Fig. 7:**
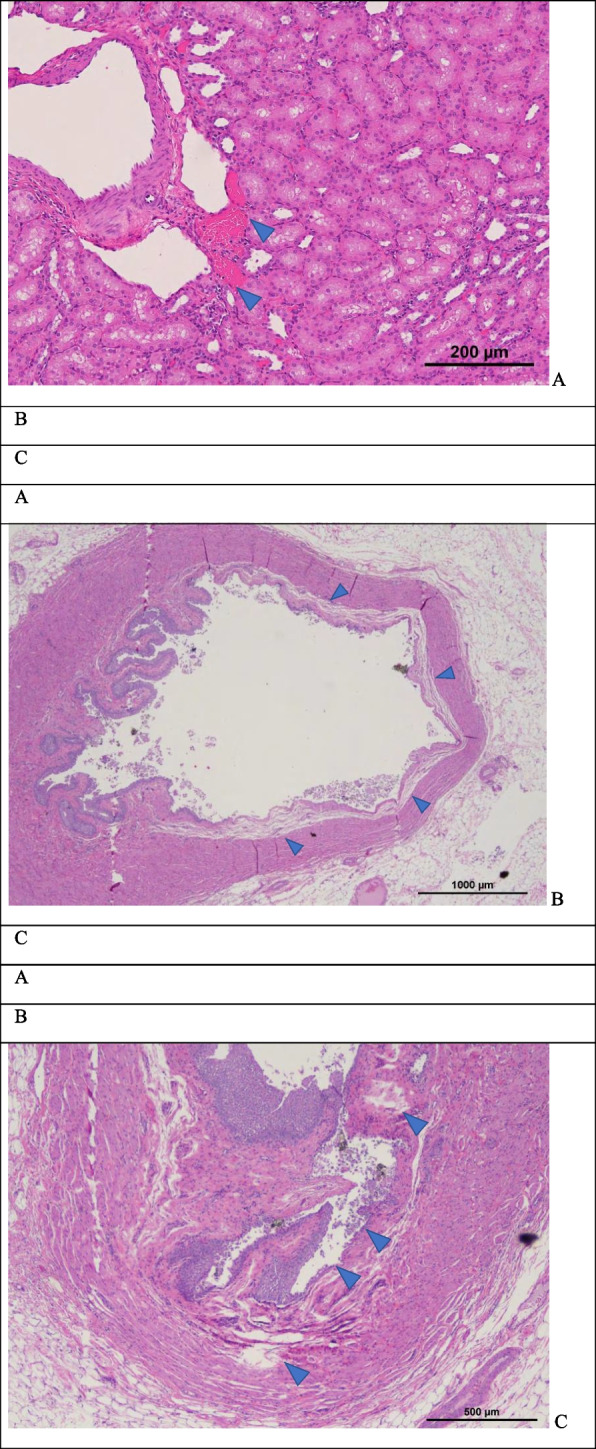
**A** Localized bleeding in renal cortex. Total damage extends approximately 200 µm × 100 µm and is consistent with mechanical trauma due to ultrasound. **B** Partial dissection of the inner layer of the ureter approximately 200 µm thick. This may be from the ultrasound exposure, but the lack of any bleeding is inconsistent with previously observed injury in vivo. It may also be from stone implantation or extraction during necropsy, or histologic processing artifacts. **C** Focal damage and localized denudation of urothelium. Focal damage is ≤ 400 µm from the urothelial surface. Stone fragments (black) are evident in the lumen. Again, the lack of bleeding is inconsistent with previously observed mechanical injury due to ultrasound exposure. Effects are potentially from stone implantation or histologic processing artifacts

There was no evidence of gross injury in the kidneys or ureter. In one of the kidneys, there were regions of focal injury in the sinus fat and renal cortex as evidenced by bleeding (Fig. 7A). The largest region of injury was around 600 $\mu$ m. All other focal injuries were smaller than 200 $\mu$ m. In the ureter, there was partial sloughing of the epithelium and in regions this extended to the lamina propria (Fig. 7B). Some focal regions of damage were also observed in the muscularis (Fig. 7C). However, no hemorrhaging was observed.

## Discussion

This article described the design and testing of a BWL platform to treat ureteroliths in cats. Obstructing ureteroliths present a serious risk to renal function, as the backpressure to the pelvis can lead to severe damage if not addressed promptly [1, 7]. In small pilot trials using extracorporeal SWL in cats, improvement in azotemia was observed even in cases where no apparent stone fragmentation was detected, indicating even small changes to the stone may partially relieve obstruction [33, 34]. However, SWL is not advised for treatment in cats, as the fragmentation success is low [34, 35]. If demonstrated effective and safe, BWL could substantially improve the approach to managing obstructing ureteroliths and other stones in cats.

The studies herein demonstrate that BWL can effectively fragment feline calcium oxalate urinary stones in vitro. BWL has been primarily developed for human application and recently reported results in 19 patients [18], but there are several differences between humans and cats that necessitate adaptation of the system. First, the human ureter is approximately 3 mm intraluminal diameter and passes stones with high probability for those < 5 mm [36]. Many BWL studies have been performed at 350 kHz where nearly all fragments in calcium oxalate monohydrate stones have been found to be < 2 mm [19, 20, 37]. The feline ureter is much smaller than humans, and data suggests that fragments should be < 1 mm to maximize success [4, 11, 22]. To adjust to this difference, we exploited a unique characteristic of BWL to control the size of fragments generated, and it has been shown possible in vitro to ‘dust’ human stones to < 1 mm size with higher frequency [19]. The frequency for the present study was designed to be 700 kHz, but we found the transducer to operate at 650 kHz most efficiently. A higher frequency exposure can break smaller stones and produces smaller fragments but requires a longer time to achieve complete fragmentation in larger stones. We aim to investigate next whether a higher-frequency device would be beneficial to treating smaller stones in cats. These design methods could also be valuable in future work translating BWL to the anatomy for other cases such as pediatric human stones or canine stones.

CT analysis indicated that nearly any location along the urinary tract had an available acoustic window in the cat from the posterior flank or abdomen. The change in transducer geometry as well as focal depth minimized the volume of the focus. These changes limited the depth of field of the focus to minimize high-amplitude ultrasound exposure to surrounding organs. In practice, ultrasound imaging will also need to be performed preprocedure to ensure no sensitive organs such as intestines are present in the high-amplitude focal region. In addition, the small skin-to-stone distance required a small focus to avoid focal effects to the skin surface from cavitation. However, it is known that for both SWL and BWL, the width of the focus should be similar to or larger than the width of the stone [15, 38–40]. Obstructing ureteroliths in cats are commonly found to be 1–4 mm [41], thus the tradeoff was limited to treating stones in this range. Nonetheless, stones can be larger than 5 mm and these would likely be difficult to design for given the constraints and tradeoffs mentioned above.

All the stones treated in this study were composed of 100% calcium oxalate monohydrate. We did not test for the response of different stone compositions to the treatment, but a large majority of upper tract stones in cats are found to be calcium oxalate [42], and of those, calcium oxalate monohydrate is the most common type. Thus, fragmentation will possibly be more consistent than in humans, where several compositions are common. Another limitation of the model applied here was that it did not strictly represent an obstructing stone as it was positioned in a way that was open to the water bath rather than surrounded by the tissue of the ureter wall. Such mechanical differences may also impact fragmentation [43].

Previous investigations have demonstrated 350-kHz BWL can be applied with pressure amplitudes up to 7 MPa to porcine kidneys with almost no discernable injury [20]. Indeed, only minor focal damage was observed when the kidneys and ureters of pigs were treated with stones implanted, and no untreated controls were performed to assess if any of the injury was associated with the surgical implantation of the stone. It is possible that the focus was not perfectly aligned with the stone since we relied on visual and auditory cues for alignment. However, no injury was found tissues adjacent to the stone. The primary injury mechanism in lithotripsy is inertial cavitation [44, 45], which occurs from the tensile half cycles of the wave oscillating microscale bubbles in the tissue. A common metric for the potential for cavitation is the mechanical index (MI) [46], for which the MI = 11.8 for a 7 MPa, 350-kHz beam. The 650-kHz exposure at a maximum pressure of 8.9 MPa for the cat transducer results in an MI = 11.0, indicating a similar potential for cavitation to those found safe in a pig model. An additional safety measure for BWL is that cavitation is readily detected by ultrasound imaging [30], and the exposure can be temporarily terminated if detected to minimize ultrasound-related injury. With these data and additional measures, we anticipate that this transducer and BWL exposure can be safely delivered to a stone in vivo, and we plan to further evaluate this technology through future clinical testing.

## Conclusions

A BWL system for noninvasive fragmentation of obstructing ureteroliths in cats was developed and tested in vitro. The transducer geometry and exposure parameters were designed to account for feline anatomic constraints common in clinical cases. Testing indicated natural calcium oxalate stones can regularly be fragmented to pieces smaller than 1 mm. These results indicate this system may be effective at fragmenting stones and relieving urinary obstruction.

## Data Availability

The datasets used and/or analyzed during the current study available from the corresponding author on reasonable request.
